# Proton MR Spectroscopy of Pediatric Brain Disorders

**DOI:** 10.3390/diagnostics12061462

**Published:** 2022-06-14

**Authors:** Stefan Blüml, Alexander Saunders, Benita Tamrazi

**Affiliations:** Department of Radiology, Children’s Hospital Los Angeles and University of Southern California Keck School of Medicine, Los Angeles, CA 90027, USA; saundera@usc.edu (A.S.); btamrazi@chla.usc.edu (B.T.)

**Keywords:** pediatrics, brain disorders, in vivo MR spectroscopy

## Abstract

In vivo MR spectroscopy is a non -invasive methodology that provides information about the biochemistry of tissues. It is available as a “push-button” application on state-of-the-art clinical MR scanners. MR spectroscopy has been used to study various brain diseases including tumors, stroke, trauma, degenerative disorders, epilepsy/seizures, inborn errors, neuropsychiatric disorders, and others. The purpose of this review is to provide an overview of MR spectroscopy findings in the pediatric population and its clinical use.

## 1. Introduction

The purpose of this article is to introduce in vivo MR spectroscopy (MRS) and its opportunities for clinical applications in pediatric patients. More comprehensive information about the various uses of MRS for pediatrics can be found elsewhere [1].

MR spectroscopy, also known as nuclear (N)MR spectroscopy, had been established before MR imaging as an analytical tool for physicists, chemists, and biochemists for the non-destructive chemical analysis of often very small samples. With the development of large and powerful magnets, (N)MR evolved dramatically to eventually allow the examination of metabolism in humans in vivo. Similar to MR imaging, MR spectroscopy is non-invasive and harmless and, thus, particularly appropriate for applications in pediatrics where exposure to ionizing radiation or radioactive isotopes is undesired.

Proton-(^1^H; hydrogen) MR spectroscopy (^1^H-MRS) uses the same hardware as MR imaging and is a “push-button” application that is widely available on state-of-the-art clinical MR scanners. Other nuclei have been utilized for in vivo MRS, including phosphorous-31 (^31^P), carbon-13 (^13^C), or fluorine-19 (^19^F). However, this report will focus on ^1^H-MRS since those more “exotic” spectroscopy methods require additional hardware and are only available at academic centers.

**Figure 1:**
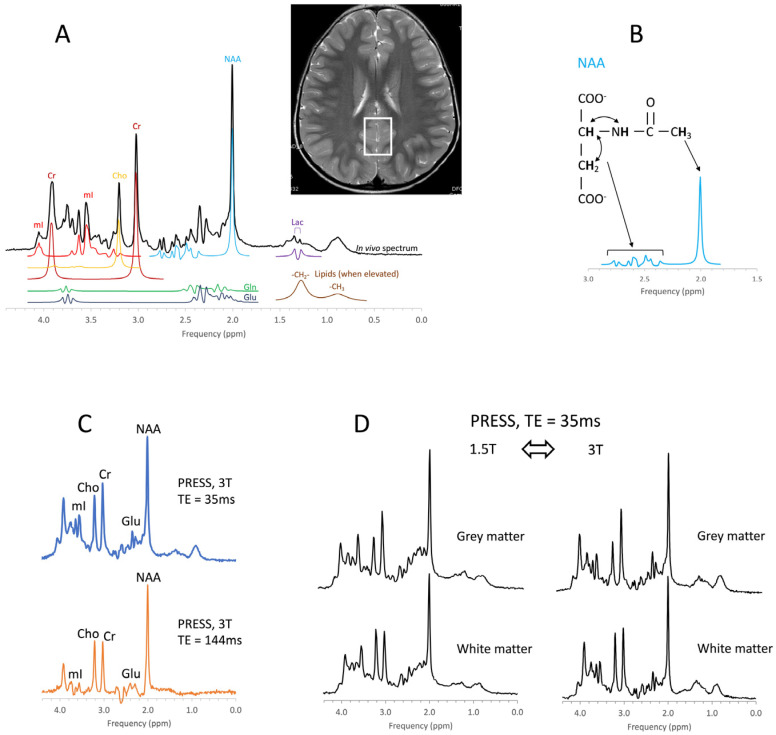
Principles of in vivo MR spectroscopy. An in vivo MR spectrum of parietal gray matter with simulated signals of some of the chemicals that typically contribute to spectra is shown (**A**). Note that signals of chemicals depend on the position of the protons within molecules and their interaction with each other. For example, the prominent signal of the N-acetylaspartate (NAA) molecule at approximately 2.0 ppm is generated by three equivalent protons of the -CH_3_ (methyl) group, while a more complex signal is generated by interacting protons elsewhere in the molecule (**B**). The position on the frequency axis and the signal pattern identifies chemicals, whereas the amplitude (area) is proportional to the concentration. Because the concentrations of these chemicals are much lower than the water content of the tissue, MRS is restricted to regions of interest (ROIs) that are much larger than the resolution of MR images (the ROI is indicated as rectangular box on the MR image). The MR signal of chemicals also depends on the acquisition method (**C**) and field strength (**D**). For example, the two spectra in **1C** represent the same metabolism with the different appearances as a consequence of the different echo times (TE). Cr = creatine, Cho = choline, mI = myo-inositol, Glu = glutamate, Gln = glutamine, Lac = lactate.

In contrast to MR imaging, which generates maps that reflect the distribution and properties of protons of water molecules (H_2_O), ^1^H-MRS combines the signals generated from the protons attached to other chemicals and produces a “spectrum” representative of tissue biochemistry (Figure 1A,B). Chemicals detectable by MRS (Table 1) are small, mobile, and mostly *intracellular metabolites*, whereas large immobile macromolecules and phospholipids, myelin, proteins, RNA, and DNA are rendered “invisible” to MRS. Synthesis and breakdown of the small amino acids, carbohydrates, fatty acids, and lipids that contribute to cell metabolism is closely controlled by enzymes, and their concentrations are, thus, kept close to constant. Therefore, the MR spectra of normal in vivo brain biochemistry are remarkably robust and comparable across subjects and serially in individuals with no “Monday morning” vs. “Friday afternoon” metabolism. Of note, to minimize motion artifacts, pediatric patients are often examined under anesthesia. We believe that the impact of anesthesia on the metabolic profiles obtained by MRS is small and likely negligible from a clinical standpoint. Yet, as definitive studies in pediatric controls are ethically not feasible, it cannot be ruled out categorically. However, the biochemical profiles vary with brain regions (e.g., white matter vs. cortical gray matter vs. deep gray matter vs. etc.) and with brain maturation—which is important to consider when interpreting pediatric MRS. Abnormal spectra will be obtained when there are structural damages (trauma, degenerative diseases, gliosis, etc.), altered physiological conditions (essentially abnormal/interrupted blood flow), tumors, or abnormal underlying biochemical and genetic conditions. A limitation of MRS is its low sensitivity. When compared with water and the water signal used for MR imaging, the concentrations of the chemicals detectable by MRS are small and generate small signals. Thus, biological/medical questions that can be appropriately addressed with MRS are “global/systemic” or “millions of cells” events in which the signal from comparably large volumes of tissue (≈1–10 cc) is analyzed.

For clinical applications of MRS, there is also a need to use consistent acquisition methods, because the appearances of MR spectra acquired from the same region of interest depend on acquisition parameters, such as the echo time (TE), and the field strength of the magnet (i.e., 1.5T vs. 3T), which could confuse interpretation (Figure 1C,D).

### MR Spectroscopy in Pediatrics

Generally, MRS in pediatrics is more challenging but also offers more opportunities than MRS in adults [2]. In the adult brain, stroke, different forms of dementia, and brain tumors (mostly astrocytomas) include most of the abnormalities that are encountered. In pediatrics, there is a wider range of diseases, which contain biologically heterogeneous tumors originating from different cell types and a wide variety of inborn errors. Furthermore, ongoing brain development adds complexity to recognizing and quantifying injury/disease as well as to prognostication.

## 2. Methods for Clinical Spectroscopy

With robust and fully automated MRS available on practically all clinical MR systems, obtaining MR spectra merely requires the selection of an appropriate acquisition method and the identification of a proper voxel or *region of interest* (ROI). Good ROI placement—and skillfully managing pediatric patients to minimize patient movements during a scan—is possibly more significant for acquiring high-quality spectra than understanding the technical details of the various MRS acquisition methods. Except for lesions, ROIs are best selected consistently in brain regions where normal in vivo metabolic profiles have been established. When examining lesions such as tumors, bleeds and calcifications should be avoided, and cellular areas should be selected over necrotic parts.

*Single-voxel* (SV) point-resolved-spectroscopy (PRESS) and stimulated echo acquisition mode (STEAM) have been the most utilized acquisition methods for clinical applications of MRS. With these methods, a single spectrum can be acquired in as little as 3–5 min from a selected brain region. Both methods can be combined with chemical shift imaging (CSI), also referred to as *multi-voxel* spectroscopy, as an alternative approach with multiple spectra acquired simultaneously from a larger volume of tissue. However, since MR scanners must find compromises when optimizing acquisition conditions for larger volumes, such as water suppression and field homogeneity, individual CSI spectra may not reach the quality of single-voxel spectra. Because of the need for spatial encoding, individual CSI acquisitions may take 10–20 min, with a higher risk for patient motion and possibly compromised data in pediatric patients who are not under anesthesia. Furthermore, CSI requires more effort and expertise for processing and review.

While clinical applications of MRS are dominated by the above-mentioned, widely available methods, there is an overabundance of other sophisticated MR spectroscopy methods that have been developed and used in academic environments. These methods may offer considerable advantages when used by skilled MR spectroscopists to answer specific biological/medical questions [3].

## 3. Metabolic Maturation of the Human Brain

From in utero to birth to adolescence, the human brain undergoes dramatic changes in size, morphology, and function. In utero, brain metabolism is tasked with providing the building blocks for the de novo synthesis of tissue. At birth, metabolism transitions into facilitating myelination and then, predominantly, providing the energy for neurotransmission and higher brain function. It is, therefore, not surprising that metabolic profiles evolve considerably, in parallel with brain development, as reported by several groups [4,5,6,7,8,9,10,11,12].

**Figure 2:**
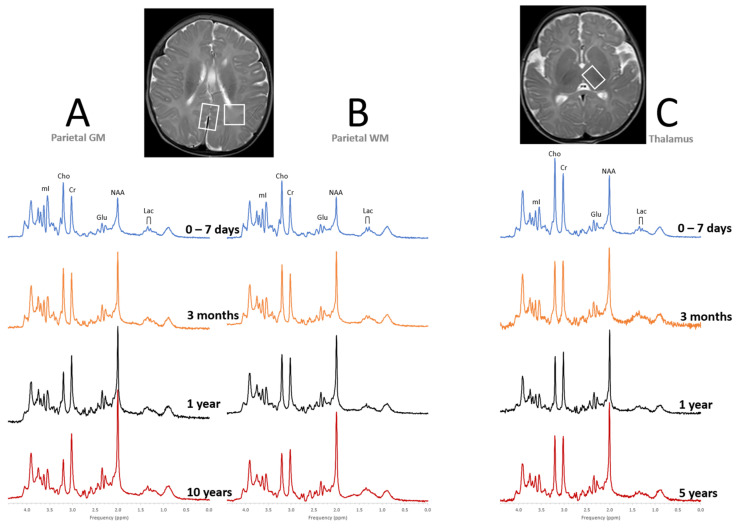
Metabolic maturation of the human brain. Shown are representative “closest-to-normal” spectra for parietal gray matter (GM, **A**), parietal white matter (WM, **B**), and thalamus (**C**) acquired from pediatric term-born patients at different points of brain development. MR images as well as clinical follow up were unremarkable for all subjects. Note, that metabolic profiles vary both with age and tissue type. All spectra were acquired on 3T scanners with SV-PRESS, echo time (TE) = 35 ms, and repetition time (TR) = 2000 ms.

Having a good understanding of normal age-dependent metabolic profiles is a prerequisite for clinical applications of MRS, particularly for the very young patients. The first three months of life is a period of rushed axonal growth, synapse formation, myelination, and neuronal maturation. During that time, concentrations of N-acetylaspartate (NAA), creatine (Cr), and glutamate (Glu) increase rapidly in parieto/occipital gray matter (GM) and parietal white matter (WM). In the thalamus, where maturation including myelination precedes cortical development, Cr levels are close to constant and more modest increases of NAA and Glu are observed during the first three months of life (Figure 2). 

NAA, essentially unknown before the arrival of in vivo MRS, is an amino acid that is mainly stored in adult-type neurons and axons. NAA is probably the most reliable non-invasive neuronal/axonal marker as it is generally believed that its concentrations correlate with axonal outgrowth and neuronal maturation. NAA is synthesized in the mitochondria of neurons, travels along axons, and is broken down in oligodendrocytes. It is a major source of acetyl groups for lipid and myelin synthesis and may also have other functions possibly depending on the maturational stage of the brain [13,14,15,16]. Creatine and its functions have been well known before the introduction of in vivo MRS. It is synthesized in the liver and kidneys and enters brain cells via the blood stream and the plasma membrane Cr transporter, where it functions as high-energy buffering system maintaining cellular ATP levels [17]. Glutamate is the most abundant excitatory neurotransmitter and is utilized in all brain regions for various brain functions [18].

In parallel with increasing concentrations of NAA, Cr, and Glu with age, myo-inositol (mI) decreases rapidly in parietal WM and GM and more gradually in the thalamus. mI is an osmolyte [19,20] and a marker of glial cells [21,22] that is either synthesized by the kidneys or can be taken up from the diet. In the context of early brain development, its role as a precursor of phosphatidylinositol (PtdIns), a membrane phospholipid important for signal transduction events [23] that is present in the white matter prior to active myelination [24,25], might be most significant. Choline (Cho) levels remain constant or decrease slowly depending on the brain region. Choline-containing metabolites (mainly phosphocholine and glycerophosphocholine) are involved in cell membrane synthesis and breakdown [26], and Cho is generally elevated in proliferating tissue.

Tissue lactate (Lac) levels are important indicators for pathology when elevated. In contrast to adults and older children, in whom lactate is barely measurable, in the normal newborn brain, lactate is expected to be detectable. Lactate levels then decline as the brain completes its transition to aerobic metabolism [8].

After around three months of life, the rate of metabolic maturation of the brain slows down considerably. Brain metabolism then stabilizes at approximately at 5 years of age with the brain reaching around 90% of adult size, albeit there are small adjustments in parallel with continuing brain development into young adulthood [11].

### Prematurity

The metabolic profiles are, in a very good approximation, functions of the post-conceptional age (PCA) [10]. That is, brain metabolism in a 34-weeker examined at 6 weeks is comparable with the metabolism of a term (40 weeks) newborn studied at birth. However, with significant changes occurring with birth that affect metabolism, such as the delivery of higher oxygenated arterial blood to the brain, irrespective of the gestational age, it is conceivable that there are slightly different trajectories of metabolic maturation in premature-born infants [27].

## 4. Clinical Applications of MR Spectroscopy

### 4.1. Pediatric Brain Tumors

Pediatric brain tumors are the second most frequent malignancy of childhood (after leukemia) with approximately 2500 new diagnoses per year in the United States. They are the leading cause of death from cancer in pediatric oncology [28,29]. Furthermore, survivors often have severe neurological, neurocognitive, and psychosocial sequelae. In contrast to adult brain tumors, which are mostly astrocytomas, pediatric brain tumors originate from different cell types and are, thus, biologically more heterogeneous with different biochemical and metabolic features.

**Figure 3:**
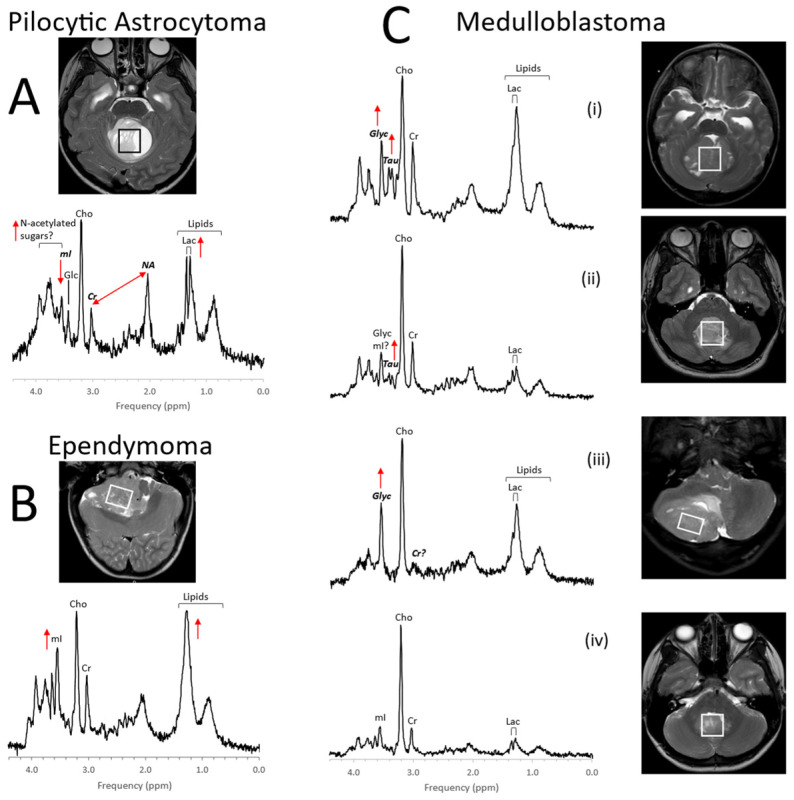
MR spectra of common pediatric posterior fossa tumors. Metabolic profiles of posterior fossa pilocytic astrocytoma (**A**) are generally relatively predictable. Pilocytic astrocytomas show elevated lactate and lipids. There is signal consistent with N-acetylated sugars (N-acetyl (NA) at ≈2 ppm and a broad signal from sugars at ≈3.8 ppm), while creatine (Cr) and myo-inositol (mI) levels are low. Ependymomas (**B**) have less predictable profiles. Whereas lipids are often prominent, they are not elevated in every ependymoma. Similarly, levels of other metabolites, such as mI can vary considerably. Medulloblastomas (**C**) are embryonal tumors that can present with strikingly different metabolic profiles for individual patients. To what extent metabolic profiles correlate with the molecular subgroups is an area of active research [41]. Above, examples for group 3 (i), group 4 (ii), sonic hedgehog (iii), and WNT (iv) are shown. Taurine (Tau) and glycine (Glyc) are often (but not always) detectable in these tumors. Medulloblastomas are generally more cellular tumors with higher absolute metabolite levels. For example, average choline (Cho) levels are approximately medulloblastoma:ependymoma:pilocytic astrocytoma = 5:3:2 [37], which cannot be appreciated when spectra, that are scaled to their tallest peaks, are compared. All spectra were acquired on 3T scanners with SV-PRESS, TE = 35 ms, and TR = 2 s.

**Figure 4:**
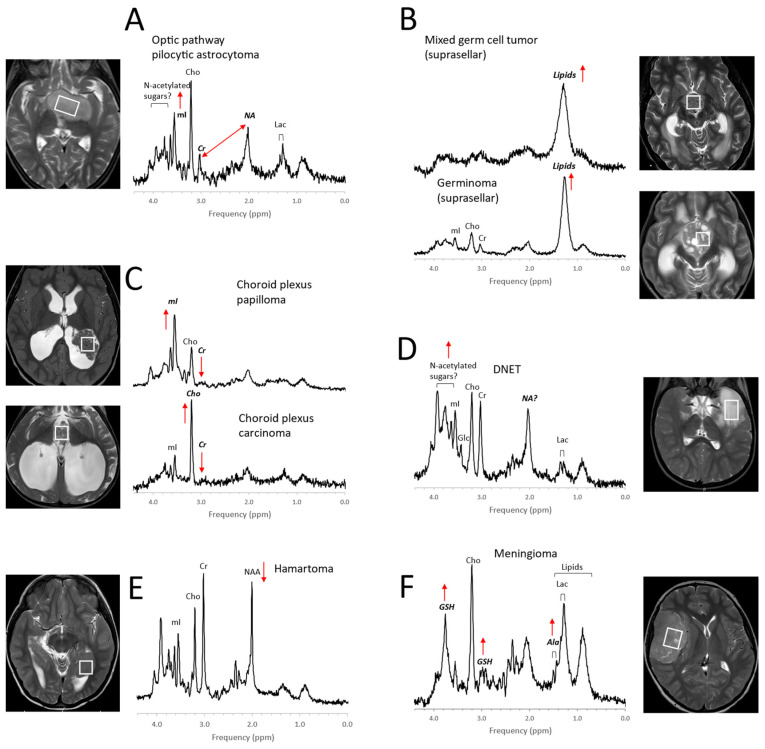
Examples for MR spectra of pediatric brain tumors outside the posterior fossa. Metabolic profiles of pilocytic astrocytomas elsewhere in the brain (**A**) are comparable with those in posterior fossa PAs, except that, often, higher myo-inositol (mI) is observed [42]. The MR spectra of germ cell tumors, including pure germinomas, (**B**) frequently show prominent lipids, and their quality is often limited (broad signals) possibly due to calcification and heterogeneity at microscopic levels. Among choroid plexus tumors (**C**), papillomas present regularly with prominent myo-inositol (mI), whereas choline (Cho) is prominent in carcinomas. Dysembryoplastic neuroepithelial tumors (DNETs, (**D**)) are low-grade glioneuronal tumors. Note that the signal at ≈2 ppm (with a corresponding broad signal at ≈3.8 ppm), is more similar (position on ppm axis and line width) to the N-acetyl (NA) signal observed in pilocytic astrocytoma than to N-acetylaspartate (NAA) in normal brain. A noncancerous hamartoma (**E**) shows a spectrum that is consistent with a mixture of tumor cells with normal tissue with only slightly reduced NAA and unremarkable lipids and lactate (Lac) as well as unremarkable other metabolic features. The presence of alanine (Ala) and high glutathione (GSH) [43,44] identifies meningiomas (**F**), a dural-based tumor. In addition, creatine (Cr) is depleted in meningiomas, and lactate and lipids are readily detectable. All spectra were acquired on 3T scanners with SV-PRESS, TE = 35 ms, and TR = 2 s.

**Figure 5:**
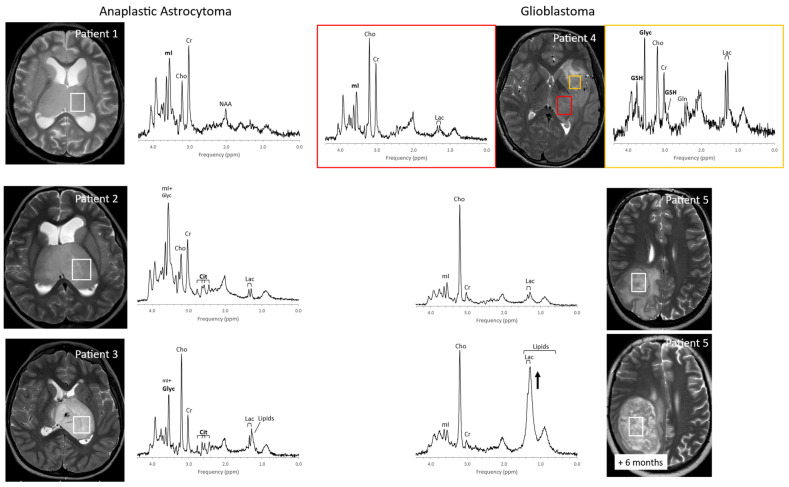
Pediatric high-grade gliomas. Metabolic profiles of pediatric high-grade gliomas present with considerable heterogeneity. For example, three patients with thalamic anaplastic astrocytoma show varying levels of myo-inositol (mI) and glycine (Glyc) at initial diagnoses. Choline (Cho) is moderate or even low in patients 1 and 2 but is prominent in patient 3, with higher Cho generally associated with more proliferative tumors [45]. Citrate (Cit) is readily detectable in patients 2 and 3 but absent in patient 1. Two spectra acquired from patient 4 (glioblastoma) at diagnosis exhibit remarkable metabolic heterogeneity with glutathione (GSH), Glyc, and lactate (Lac) all elevated in one region but unremarkable in a second spectrum. It is presently unclear to what extent metabolic features identify subtypes and whether this information can be exploited to optimize therapeutic approaches and patient management. Serial MRS in patient 5 (glioblastoma) demonstrate the transition of a solid lesion to a partially necrotic lesion with increased lipids and lactate (Lac). All spectra were acquired on 3T scanners using SV-PRESS, TE = 35 ms, and TR = 2 s.

Initial MRS studies have focused on common infratentorial tumors (medulloblastomas, pilocytic astrocytomas, ependymomas) and reported that proton MRS can be used to help differentiate cerebellar tumors by looking at ratios of NAA, creatine, choline, and lactate [30]. Since then, several groups [31,32,33,34,35,36,37,38,39], taking advantage of the availability of robust short echo-time (TE) MRS, have independently confirmed the value of in vivo MRS for improving the accuracy of initial diagnoses including for tumors outside the posterior fossa such as germ cell tumors, choroid plexus tumors, and high-grade gliomas (Figure 3, Figure 4 and Figure 5). It should be noted that high-grade gliomas in pediatrics are a heterogeneous group of tumors that are biologically different from high-grade gliomas in adults [40], and significant metabolic heterogeneity can be observed across subjects, in individual patients in different areas of the lesion, and in serial studies.

**Figure 6:**
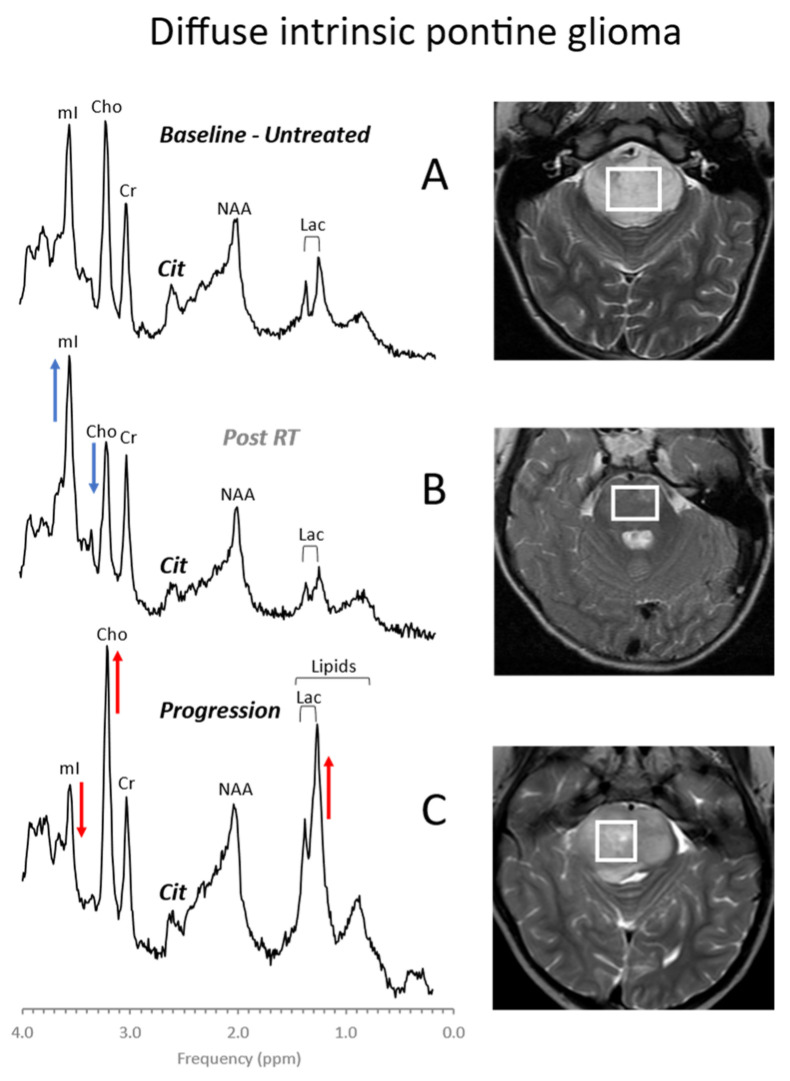
Diffuse intrinsic pontine glioma (DIPG). DIPGs are readily diagnosed by conventional MR imaging. MR spectroscopy demonstrates a metabolic evolution from a more moderately abnormal profile at presentation (**A**) to a metabolic pattern that is consistent with high-grade aggressive behavior at progression (**C**). Transiently, albeit still consistent with viable tumor, a pattern suggestive for a limited response to therapy may be observed, characterized by reduced choline (Cho) and increased myo-inositol (mI) (**B**). Metabolic changes consistent with progression may precede clinical deterioration and progression on MRI. All spectra were acquired on a 1.5T scanner using SV-PRESS with TE = 35 ms and TR = 1.5 s. Cit = citrate.

Among pediatric brain tumors, diffuse intrinsic pontine gliomas (DIPGs) carry the worst prognosis. They are highly resistant to chemo- and radiation therapy and, due to their location in the brainstem, inoperable. Thus, with no effective therapy available, the average survival after diagnosis is less than one year [46]. In vivo MR spectroscopy studies showed that, at initial diagnoses, these tumors often present with metabolic profiles that are consistent with low-proliferative tumors. The metabolism of DIPG then evolves into a profile typical for high-grade gliomas consistent with the observation that, at autopsy, most DIPGs have progressed to glioblastoma (Figure 6) [47,48]. These changes may precede clinical deterioration and progression on MRI, and MR spectroscopy could thus provide non-invasive biomarkers that help with patient management [49].

Citrate (Cit) is routinely detectable in DIPG. Citrate is also detectable in subgroups of tumors outside the brainstem [50]. Among grade II astrocytomas, high levels of Cit appeared to indicate a high risk for malignant progression [51]. However, Cit was not generally specific for poor outcome, as it was undetectable in a significant number of high-grade gliomas with poor outcomes. Harris et al. reported that myo-inositol in supratentorial pilocytic astrocytomas is higher than in posterior fossa pilocytic astrocytomas (cf. Figure 3 and Figure 4). They also noted that, among optic or thalamic tumors, those that had low myo-inositol at presentation were at higher risk for progression [42]. It has also been suggested that elevated levels of glycine identify tumors with increased malignancy [52,53,54].

Recently, molecular subtypes of common pediatric brain tumors associated with significant different clinical outcomes have been identified using whole-genome sequencing methods [55,56]. Future novel targeted therapies might be able to treat these tumors without the need and risks of surgical resection and biopsies. Nevertheless, there is a need for accurate and early in vivo diagnosis of molecular subtypes—a possible important clinical application for in vivo MRS. Indeed, first studies indicate that MRS might be able to assist with the non-invasive identification of the medulloblastoma subtypes wingless (WNT), sonic hedgehog (SHH), group 3, and group 4 [41]. In vivo MRS may also predict key molecular features of atypical teratoid/rhabdoid tumors (AT/RT) at initial diagnosis [57] and may help with assigning subtypes of ependymomas [58].

### 4.2. Perinatal Hypoxic–Ischemic Encephalopathy

**Figure 7:**
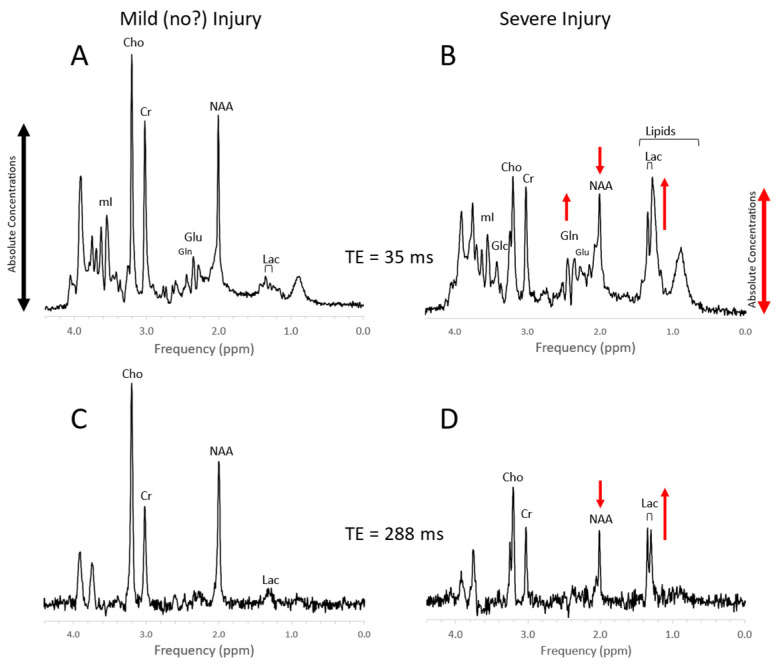
Newborn hypoxic/ischemic injury. Typical MR spectra of the thalamus of acute mild newborn hypoxic/ischemic injury (HIE) with clinically unremarkable follow up (**A**,**C**) versus severe HIE followed by death or significant disability (**B**,**D**). Note that spectra (**A**) + (**B**) were acquired with a short echo time (TE = 35 ms), whereas spectra (**C**) + (**D**) were acquired with long TE = 288 ms. Metabolic markers of severe injury that have been consistently reported in the literature are elevated lactate (Lac) and lipids, reduced NAA, and elevated glutamine (Gln). The above spectra were scaled to the approximate absolute metabolite levels ((**A**) vs. (**B**) and (**C**) vs. (**D**)). Edema formation and/or cell death and depletion of intracellular metabolites in severe HIE may explain generally lower absolute concentrations. In long-TE spectra, signals from lipid, glutamate, and glutamine are suppressed resulting in a more unambiguous detection and quantitation of NAA and lactate, which may simplify the determination of the important Lac/NAA ratio. Spectra were acquired within 1 week of injury on a clinical 3T scanner with SV-PRESS, TR = 2 s, and TE as indicated above.

Perinatal hypoxic–ischemic encephalopathy (HIE) is a significant cause of neonatal death and of long-term neurodevelopmental disabilities [59]. MR imaging of the newborn brain provides biomarkers of disease status and predictors for outcome [60,61,62,63]. MRS complements conventional MRI and diffusion MRI by providing direct measures of metabolites that reflect the severity of injury [63,64,65,66,67,68,69,70] (Figure 7). Meta-analyses that compared various imaging modalities showed high sensitivity (82%) and specificity (95%) for the lactate to NAA ratio (Lac/NAA) for predicting neurodevelopmental outcomes [71,72]. Lactate accumulates when oxidation of pyruvate in the TCA cycle is impaired or halted, whereas a reduction of NAA indicates neuronal and axonal injury.

**Figure 8:**
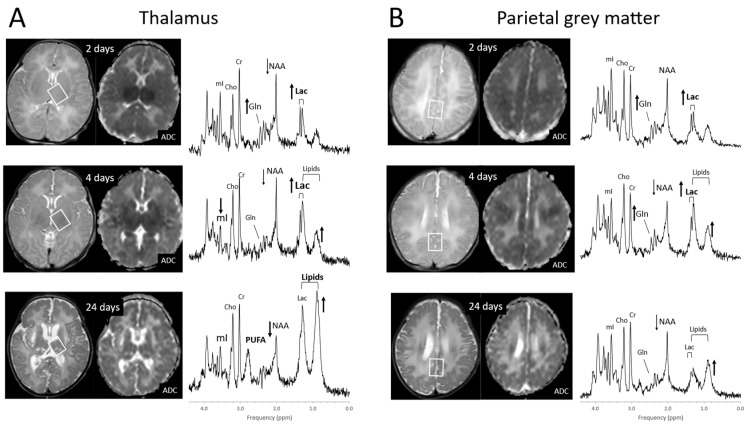
Evolution of HIE in newborns. MR spectra of the thalamus (**A**) and parietal gray matter (**B**) in a newborn with severe HIE on days 2, 4, and 24 after injury. Lactate (Lac) is elevated whereas lipids are unremarkable on day 2 after injury in both brain regions. Four days after injury, small increases of lipids are noted with lactate remaining elevated. Lipids are prominent at day 24 in the more severely injured thalamus. At that time, lactate levels in both regions have decreased. The broad peak at 2.8 ppm originates from poly-unsaturated fatty acids (PUFAs). Note that *absolute* NAA is reduced on day 2 but then further declines with cell death particularly in the thalamus. Spectra were acquired on a clinical 3T scanner with SV-PRESS, TE = 35 ms, and TR = 2 s.

**Figure 9:**
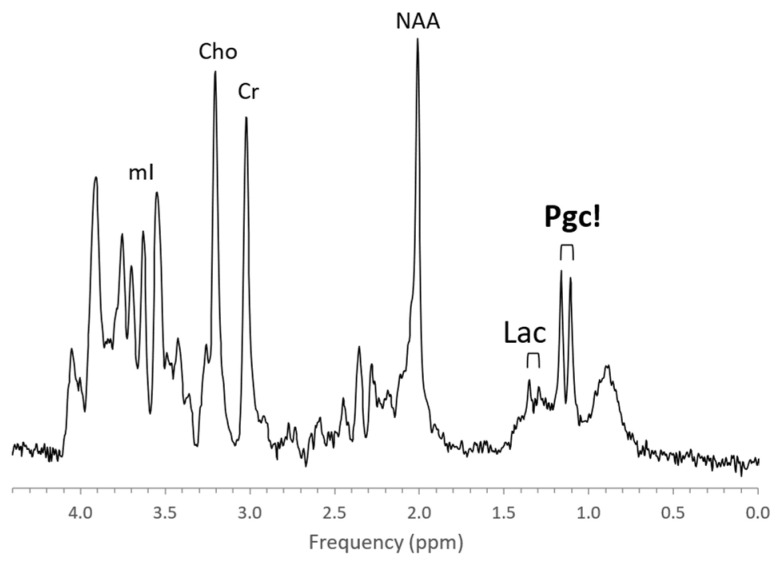
Lactate vs. propylene glycol. MRS of parietal white matter of a 1-month-old male with suspected HIE shows a prominent propylene glycol (Pgc) signal centered at ≈1.19 ppm but essentially unremarkable Lac at ≈1.33 ppm. Other metabolites are also unremarkable.

The metabolic profiles of HIE evolve as the injury evolves. Lactate is prominent and glutamine (Gln) is elevated at the very early stage (1–2 days) with NAA more moderately decreased. Edema formation may also have an overall impact on metabolite profiles and concentrations during the acute phase of HIE. With subsequent cell death in severe HIE, a more substantial reduction of NAA and increased lipids are observed (Figure 8). Kreis et al. suggested that MRS performed 3–4 days after injury had higher predictive value for outcome than when done at 1–2 days; however, their study was performed in older children [73]. Of note, propylene glycol (Pgc), when used as a vehicle for medications, can accumulate in tissue and can be misidentified as lactate as their signals are similar [74]. This is avoided by appreciating the different positions of their signals on the frequency axis at ≈1.14 ppm for Pgc vs. ≈1.33 ppm for Lac (Figure 9). Pgc is ultimately metabolized to lactate and is, therefore, a potential exogenous source of lactate.

### 4.3. Inborn Errors of Metabolism

With MRS providing metabolic information, inborn errors of metabolism (IEMs) seem to be a tailormade application for MRS. However, IEMs are generally infrequently encountered indications for brain MRI studies when compared with, for example, brain tumors. In addition, albeit altogether IEMs constitute a significant portion of childhood disorders, individually, IEMs are rare diseases. Finally, since radiologists are often uncomfortable with MRS, it has been utilized infrequently, and it is not surprising that the number of MRS studies of IEM is small. Nevertheless, MR imaging studies, for IEM patients who undergo an MR examination, are often interpreted as unremarkable or report ambiguity. For these patients, with a suspected or a known neurometabolic disease, the addition of a brief MRS acquisition may be beneficial either by improving the accuracy of diagnoses or the phenotyping of a known disease [75].

**Figure 10:**
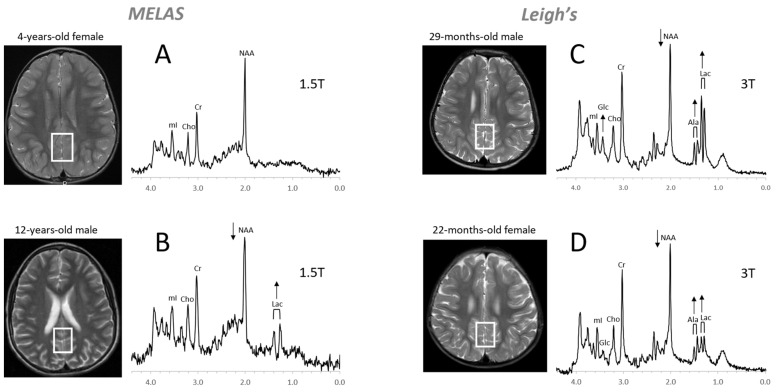
Mitochondrial disorders. MRS of parieto/occipital gray matter of mild form of MELAS (mitochondrial encephalomyopathy, lactic acidosis, and stroke-like episodes) with an unremarkable MRI is essentially normal with no evidence for elevated lactate (Lac) (**A**). In contrast, Lac is readily detectable in another MELAS patient with a borderline normal MRI. Note that NAA (relative to Cr) is reduced in this patient (**B**). MR spectra of two patients diagnosed with Leigh’s syndrome (siblings) are shown on the right. In addition to Lac, alanine (Ala) is elevated. In both spectra, NAA is reduced, and glucose (Glc) seems to accumulate in the upper spectrum (**C**,**D**). Spectra were acquired with SV-PRESS, TE = 35 ms, TR = 1.5 s on a 1.5T scanner (**A**,**B**) and TE = 35 ms, TR = 2 s on a 3T scanner (**C**,**D**).

**Figure 11:**
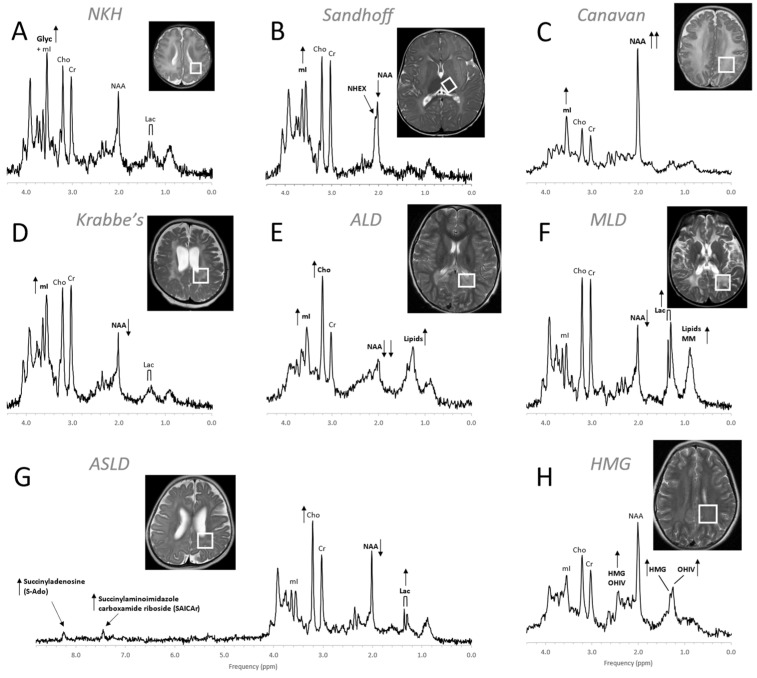
MR spectra of various inborn errors of metabolism. (**A**) Non-ketotic hyperglycinemia (NKH): A 15-day-old male newborn examined to confirm suspicion of acute NKH. The elevated glycine (Glyc) signal is consistent with hyperglycinemia, an amino aciduria in which a defect of the enzyme that breaks down glycine results in the abnormal accumulation of glycine in tissue. Note that for a 15-day-old newborn, the NAA and Lac signals are within normal. Other metabolic features are also unremarkable when adjusted for age. For the above patient, the MR images were mostly unremarkable. (**B**) Sandhoff disease: A 14-month-old female presenting with global developmental delay and hypotonia with delayed myelination and diffuse white matter abnormalities. The signal at approximately ≈2.07 ppm has been assigned to N-acetylhexosamine (NHEX), specific for Sandhoff disease [78]. In addition, elevated mI and reduced NAA is noted. (**C**) Canavan disease: Canavan disease is a leukodystrophy where a defect in aspartoacylase (ASPA), the enzyme that breaks down N-acetylaspartate (NAA), results in excessive accumulation of NAA. In above spectrum, myo-inositol (mI) is also elevated. The MR images of the 6-month-old male patient showed significant diffuse white and gray matter abnormalities. (**D**) Krabbe’s leukodystrophy: Krabbe’s leukodystrophy is a lipid storage disorder caused by a deficiency of galactocerebrosidase (GALC), the enzyme required for the breakdown of the sphingolipids, galactosylceremide and psychosine. MR images of a 3-year-old child with Krabbe’s leukodystrophy demonstrate white matter dysmyelination and loss. MRS of white matter show a significant reduction of NAA and elevated mI. (**E**) Adrenoleukodystrophy (ALD): ALD is caused by mutations in the ABCD1 genes. In vivo MRS of affected the white matter in a 5-year-old male shows, relative to creatine (Cr), elevated lipids, depleted NAA, elevated choline (Cho), and elevated mI. Note that the spectrum carries some similarities with the spectra of gliosis and gliomas. (**F**) Metachromatic leukodystrophy (MLD): In MLD the accumulation of sulfatides causes the destruction of the myelin sheath. MR images show profoundly abnormal white matter. The MR spectrum shows elevated lipids and macromolecules (MM), elevated Lac, and reduced NAA. (**G**) Adenylosuccinate lyase deficiency (ASLD): ASLD causes the buildup of succinylaminoimidazole carboxamide riboside (SAICA riboside) and succinyladenosine (S-Ado), which are detectable at 7.5 and 8.3 ppm. In addition, in this patient, Lac is elevated, NAA is reduced, and Cho is elevated relative to Cr. The MR images of the 17-month-old female showed general volume loss and hypomyelination. (**H**) 3-hydroxy-3-methylglutaric acid (HMG) CoA lyase deficiency: In HMG CoA lyase deficiency, cells are unable to process leucine and synthesize ketone bodies. The MR images of this 12-year-old female were mildly abnormal. The MR spectrum acquired in parietal white matter demonstrates accumulation of HMG and of 3-hydroxy isovaleric acid (OHIV). All spectra were acquired on clinical 1.5T (**C**,**E**,**H**) or 3T scanners (**A**,**B**,**D**,**F**,**G**) with SV-PRESS sequence, TE = 35 ms, and TR = 1.5 s (1.5T) or TR = 2 s (3T).

MR spectra of inborn errors need to be interpreted in the context of the complete clinical history and MRI appearance as there are common abnormal metabolic features such as possibly elevated lactate and glucose in mitochondrial disorders or reduced NAA for any disease associated with neuronal/axonal damage. Age at onset, progression of a disease, and treatments, particularly those that are effective (e.g., removal of glycine in hyperglycinemia), will change the appearance of the spectra [70,76,77]. Examples for MRS of inborn errors acquired at a single institution with consistent methods are shown in Figure 10 and Figure 11.

### 4.4. Trauma

Traumatic brain injury (TBI) in children, including from child abuse (non-accidental trauma), is a leading cause of child death and neurologic complications in the United States [79,80,81]. In addition, possible adverse long-term effects from mild but repeated TBI (concussions) from sports or other activities are likely underreported and are an increasing concern in children [82,83,84,85].

Computer tomography (CT) and MR imaging are the first choices for detecting bleeds and edema/swelling in acute and severe TBI. However, some aspects of acute and chronic injury or more mild but repetitive injury at a cellular level may be difficult to recognize by these methodologies. Metabolic patterns observed by in vivo MRS should be expected to be heterogeneous depending on the time after injury, the severity of the injury, the brain region examined, and the response of the brain to injury, which may vary during brain development. 

Several groups independently concluded that MRS has value when performed early after an injury as it is helpful for evaluating the extent of injury and improves the accuracy of long-term prognosis [86,87,88,89,90,91,92,93]. It was reported that abnormal MRS in brain regions that were deemed to be normal by MRI, predicted outcome more accurately than the abnormalities of lesions [94,95].

**Figure 12:**
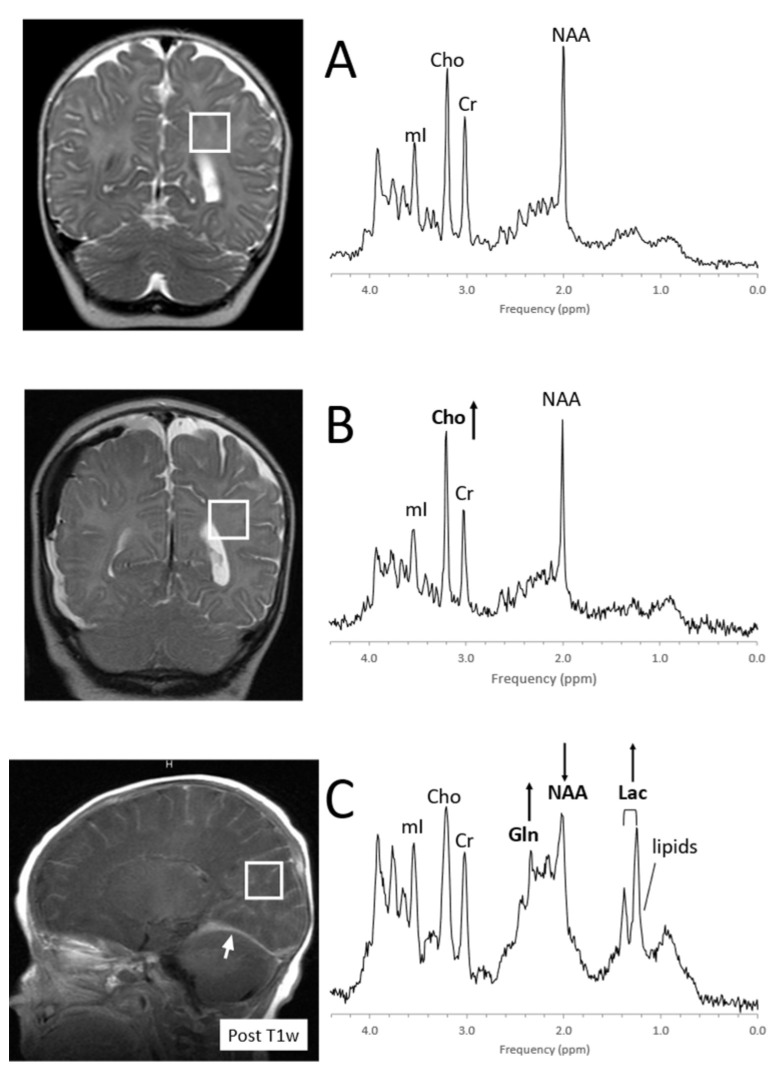
MRS of suspected non-accidental trauma. A parietal white matter spectrum acquired from a 5-month-old male with subarachnoid hemorrhage but otherwise unremarkable MR imaging. The MR spectrum appears to be normal for age (**A**). Six-month-old with subdural hemorrhages and diffuse supratentorial volume loss. Choline (Cho) appears to be elevated suggestive for axonal injury (**B**). Two-month-old with acute subdural hemorrhage in the posterior fossa and diffusion abnormality consistent with acute infarct. Lactate is elevated and NAA is reduced. The elevated signal in the 2.2–2.5 ppm range is likely from glutamine (Gln) (**C**). All spectra were acquired on a 1.5T system with SV-PRESS, TE = 35 ms, TR= 1.5 s.

Severe, acute injury is recognized by elevated lactate and lipids and a reduction of the axonal/neuronal marker NAA and often mimics MRS patterns that are observed in hypoxic–ischemic injury, consistent with the interruption of blood perfusion and subsequent apoptosis and cell death (Figure 12). In more mild/moderate cases of traumatic brain injury, NAA may be mildly or only transiently reduced, whereas Cho is often elevated, possibly reflecting axonal injury and subsequent repair processes [95,96,97,98,99].

### 4.5. Infections, Inflammation

**Figure 13:**
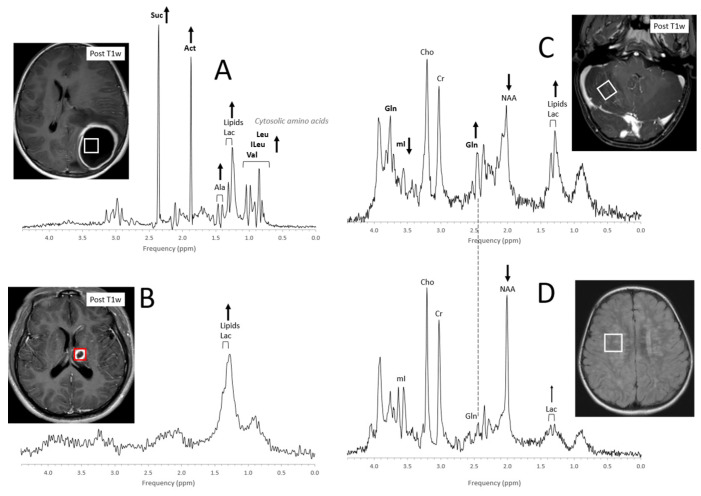
Acute and chronic infections. MR spectra of acute abscesses can be strikingly unusual. In the above example (**A**), common brain metabolites are absent, whereas prominent signals form succinate (Suc) and acetate (Act) as well the cytosolic amino acids leucine (Leu), isoleucine (ILeu), and valine (Val) are observed. lactate (Lac), alanine (Ala), and moderate amounts of lipids are also detectable. On the other hand, only lipids and lactate are observed in a shrinking abscess after 20 days of antibiotics treatment (**B**). A spectrum of acute cerebellitis shows elevated lipids and lactate as well as reduced N-acetylaspartate (NAA). Glutamine (Gln) is elevated, whereas myo-inositol (mI) is low (**C**). In a spectrum acquired from a 2 ½-year-old child with a history of meningoencephalitis, lipids are unremarkable, lactate is close to normal, and both myo-inositol and glutamine are unremarkable. NAA is reduced, indicating some permanent neuronal/axonal injury (**D**). Spectra were acquired on 3T (**A**,**C**,**D**) and 1.5T (**B**) scanners with SV-PRESS, TE = 35 ms, and TR = 2 s (3T) or TR = 1.5 s (1.5T).

Acute abscesses can present with metabolic profiles that are strikingly unusual depending on the organism (bacteria, fungi) that is causing them (Figure 13A). The presence of cytosolic amino acids (e.g., leucine, isoleucine, and valine at 0.8 ppm) has been consistently reported both in aerobic and anaerobic pyogenic lesions with varying amounts of lactate and lipids. Acetate at 1.9 ppm and succinate at 2.4 ppm may also be observed as well as other signals that yet need to be assigned [100,101,102,103]. Abscesses that have been treated successfully may present with elevated Lac and lipids with all other metabolites being depleted (Figure 13B).

Otherwise, infectious or inflammatory conditions may present with a wide range of metabolic abnormalities depending on type, time of onset, extent, and treatments [104,105,106,107,108,109,110,111,112,113]. It has been suggested that MRS could be useful for differentiating infectious/demyelinating processes from tumors by the relative prominence of glutamine in short-TE spectra [114,115]. Of note, with long-TE MRS acquisitions (e.g., TE > 130 ms), the glutamine signal disappears and spectra need to be interpreted carefully [116]. Furthermore, when the ROI for a tumor study includes edema/inflammation adjacent to a tumor, the resulting spectrum will show a combination of metabolic features, complicating the interpretation.

### 4.6. Epilepsy

Epilepsy is a chronic disorder characterized by unprovoked seizures. Seizure cause is often unknown. Several groups have reported a decrease of the neuronal marker NAA, which could be attributed to loss of neurons but also to abnormal mitochondrial function as NAA is synthesized in the mitochondria [117,118,119,120]. NAA can recover to normal levels with disappearing seizure activity [121,122]. It has also been reported that lactate is elevated during seizure activity possibly due to increased energy demand that is met with partially anaerobic metabolism [123]. Tissue glutamate and GABA levels, being major excitatory and inhibitory neurotransmitters, have been investigated by several groups in patients with epilepsy. However, it is challenging to accurately quantify these metabolites in vivo since their MR signals are complex and overlapping [124,125,126,127,128]. 

Clinically, focal abnormal metabolic features could be exploited for detecting seizure foci and for disease lateralization in temporal lobe epilepsy. Nevertheless, in practice, that would require examining the whole brain or a large section of the brain with multi-voxel spectroscopy, which is time-consuming and requires elaborate post-processing by experienced spectroscopists and is, therefore, currently not utilized.

**Figure 14:**
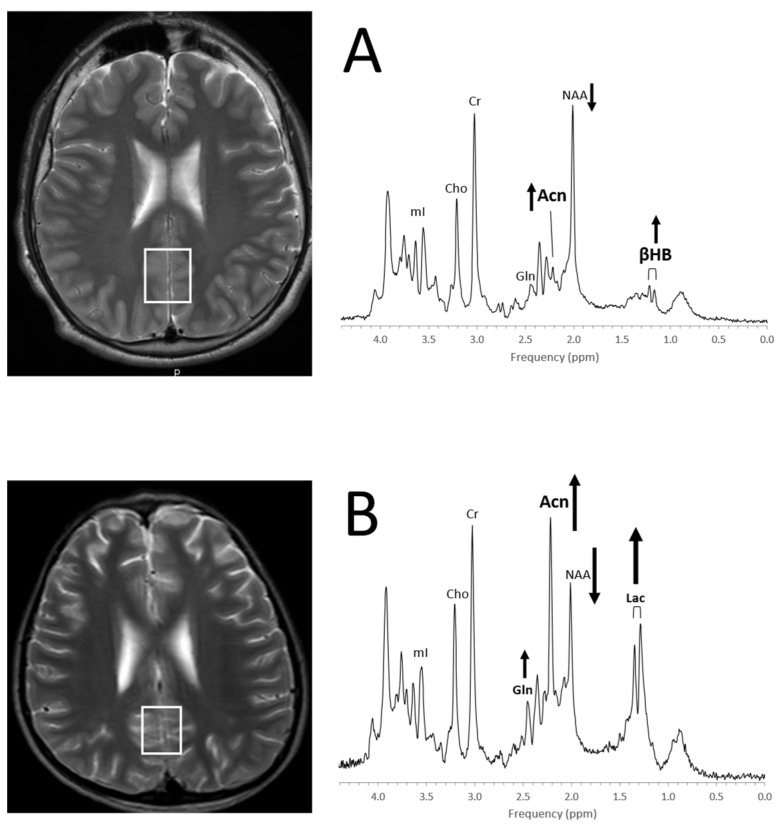
Detection of ketone bodies in in vivo MRS (**A**) The MR spectrum of a 13-year-old boy with refractory epilepsy on ketogenic diet and unremarkable MR images shows a signal consistent with acetone (Acn) at 2.22 ppm and a doublet from β-hydroxybutyrate (βHB) centered at ≈1.19 ppm. Note that NAA is below normal for age. MR imaging in this patient was unremarkable. (**B**) The MR spectrum of a 11-year-old boy with abnormal MRI, a history of meningoencephalitis, and refractory epilepsy on ketogenic diet shows prominent Acn signal, elevated lactate (Lac) and glutamine (Gln), as well as drastically reduced NAA. Spectra were acquired on a 3T scanner with PRESS, TE = 35 ms, and TR = 2 s.

A ketogenic diet is effective in reducing seizure activities for a subgroup of patients. Albeit ketosis can readily be monitored with urine ketone levels, it should be noted that the accumulation of ketone bodies in the brain can be observed with in vivo MRS [129] (Figure 14).

### 4.7. Neuropsychiatric Disorders

In vivo metabolic abnormalities have been detected in several neuropsychiatric disorders in children [130,131,132]. Unfortunately, some of the neurochemicals that are of interest for psychiatric disorders, including GABA and glutathione, cannot be quantified accurately in clinical settings. In addition, metabolites that can be assessed more reliably, such as NAA, Cr, Cho, and mI, show a large overlap in patients and controls. Thus, there is currently no role for MRS as a clinical tool for the diagnoses and monitoring of neuropsychiatric disorders in pediatrics, and MRS is restricted to academic research.

## 5. Conclusions

In vivo proton MRS is a unique and non-invasive methodology widely available on state-of-the-art clinical MR scanners. It generates spectra that contain biochemical information about tissue status. Most chemicals detectable with MRS are small molecules that maintain cell function. Analyzing metabolic profiles can increase the diagnostic accuracy and improve the assessment of disease status for a variety of pediatric brain disorders, including pediatric brain tumors, hypoxic–ischemic injuries, inborn errors of metabolism, trauma, and infections.

## Figures and Tables

**Table 1:** Metabolites detectable with clinical MR spectroscopy in the human brain.

Metabolite (Abbr.)	Functional Role and Remarks	Decreased ^a^	Increased ^a^
Acetate (Act)	Energy source, precursor of acetyl-CoA, common building block for biosynthesis	Disease correlate unknown	Infection/abscesses, brain death
Acetoacetate (AcAc)	Energy source, produced in the mitochondria of liver cells from acetoacetyl coenzyme A (CoA)	Disease correlate unknown	Ketosis
Acetone (Acn)	Produced by decarboxylation of acetoacetate, singlet at 2.22 ppm more readily detectable than βHB (see below)	Disease correlate unknown	Ketosis
Alanine (Ala)	Amino acid, protein constituent, glucose–alanine cycle	Disease correlate unknown	Inborn errors; meningioma and subgroups of other tumors
Aspartate (Asp)	Excitatory neurotransmitter NAA and Glu precursor	Disease correlate unknown	Challenging to recognize due to complex signal and signal overlap with NAA and other chemicals
β-Hydroxybutyrate (βHB)	Produced by the decarboxylation of acetoacetate, doublet similar to lactate but at 1.19 ppm	Disease correlate unknown	Ketosis
Choline (Cho) = glycerophosphocholine + phosphocholine + free choline	Membrane/myelin synthesis/degradation, acetylcholine precursor, osmolyte	Liver disease; hypo-osmotic state; during cooling (hypometabolic?)	De novo synthesis of biomass, including tumors, brain growth, tissue repair; hyper-osmotic state
Citrate (Cit)	TCA cycle intermediate, produced when the glycolytic rate exceeds TCA activity, fatty acid synthesis	Disease correlate unknown	Newborns, subgroups of tumors, most common in diffuse intrinsic brainstem gliomas
Creatine (Cr) = free creatine (fCR) + phosphocreatine (PCr)	Energy metabolism, energy storage PCr <-> fCr + ATP	Cells without creatine kinase, creatine deficiencies, some tumors	Subgroups of gliomas, gliosis?
γ-Aminobutyric acid (GABA)	Inhibitory neurotransmitter	Disease correlate unknown	Challenging to recognize due to complex signal and signal overlap with other chemicals
Glucose (Glc) (α and β isomers)	Principal fuel for cells	Hypoglycemia, detection challenging	Uncontrolled diabetes; hyperglycemia
Glutamate (Glu)	Excitatory neurotransmitter	Most tumors, hepatic encephalopathy, acute hypoxic/ischemic injury	Subgroup of seizures
Glutamine (Gln)	Part of the Glu–Gln neurotransmitter cycle; hyper ammonia detoxifier, fuel, osmolyte	Disease correlate unknown	Most tumors, edema (relative increase), demyelinating lesions, hepatic encephalopathy, acute hypoxic/ischemic injury
Glutathione (GSH)	Consists of glycine, cysteine, and glutamate. Present in reduced (predominant) and oxidized form. Marker of oxidative stress	Disease correlate unknown	Meningioma
Glycine (Glyc)	Neurotransmitter inhibitory and excitatory, cellular migration and circuit formation, antioxidant	Disease correlate unknown	Medulloblastoma and other tumors; hyperglycinemia
Lactate (Lac)	Endpoint of anaerobic glycolysis, in normal brain present in cerebrospinal fluid at higher concentrations than in tissue	Disease correlate unknown	Inborn errors of energy metabolism, hypoxic/ischemic injury; tumors, cystic lesions, normal newborn
Lipids (Lip) with contributions from macromolecules (MM)	Indicators for cell membrane breakdown when elevated	Disease correlate unknown	Injury/cell death and tumor subgroups
Leucine (Leu), iso-leucine (ILeu), valine (Val)	Branched-chain amino acids (BCAA)	Disease correlate unknown	Elevated in inborn error of BCAA metabolism, acute abscesses
Myo-inositol (mI)	Glial marker, involved in phospholipid membrane metabolism, osmolyte	Liver disease, hepatic encephalopathy, osmotic imbalance	Normal newborns, astrocytes, subgroups of tumors (e.g., astrocytoma, ependymoma, choroid plexus papilloma), osmotic imbalance
N-acetylaspartate (NAA)	Marker for mature neurons and axons	Pathologies associated with neuronal/axonal damage/loss, mitochondrial function?	Canavan disease
N-acetylaspartate glutamate (NAAG)	Neurotransmitter release modulator, small shoulder next to NAA, detectable in high-quality spectra	Disease correlate unknown	unknown
Phenylalanine	Essential amino acid	Disease correlate unknown	Uncontrolled phenylketonuria (PKU, phenylalanine hydroxylase deficiency)
Propylene glycol (Pgc)	Medication solvent (e.g., anticonvulsants), metabolizes to lactate, doublet similar to lactate but at 1.14 ppm	Disease correlate unknown	Frequently seen in newborns on medications, possibly because of underdeveloped blood–brain barrier
Scyllo-inositol (sI)	Symmetric sugar–alcohol isomer, osmolyte, inhibits amyloid-beta aggregation?	Disease correlate unknown in majority of population	Detectable under normal conditions in a subgroup of the population; glial tumors
Succinate (Suc)	TCA cycle intermediate	Disease correlate unknown	Abscesses, infection
Taurine (Tau)	Osmolyte, modulator of neurotransmission	Decreasing with normal brain maturation	Newborns; medulloblastoma (group 3, group 4), germinoma, pineoblastoma, and possibly others

^a^ The accuracy for detecting some of the metabolites is low, even if present in the tissue, due to low concentrations and/or due to complex signals that overlap with signals from other chemicals. For these chemicals, observing a reduction or even an increase is virtually impossible and unless dramatic, may be missed in individual spectra.

## Data Availability

Not applicable.

## References

[1] Blüml S., Panigrahy A. (2013). MR Spectroscopy of Pediatric Brain Disorders.

[2] Liserre R., Pinelli L., Gasparotti R. (2021). MR spectroscopy in pediatric neuroradiology. Transl. Pediatr..

[3] de Graaf R.A. (2019). In Vivo NMR Spectroscopy Principles and Techniques.

[4] van der Knaap M.S., van der Grond J., van Rijen P.C., Faber J.A., Valk J., Willemse K. (1990). Age-dependent changes in localized proton and phosphorus MR spectroscopy of the brain. Radiology.

[5] Hüppi P.S., Posse S., Lazeyras F., Burri R., Bossi E., Herschkowitz N. (1991). Magnetic Resonance in Preterm and Term Newborns: ^1^H-Spectroscopy in Developing Human Brain. Pediatr. Res..

[6] Kreis R., Ernst T., Ross B.D. (1993). Development of the human brain:In vivo quantification of metabolite and water content with proton magnetic resonance spectroscopy. Magn. Reson. Med..

[7] Toft P.B., Leth H., Lou H.C., Pryds O., Henriksen O. (1994). Metabolite concentrations in the developing brain estimated with proton MR spectroscopy. J. Magn. Reson. Imaging.

[8] Cady E.B., Penrice J., Amess P.N., Lorek A., Wylezinska M., Aldridge R.F., Franconi F., Wyatt J.S., Reynolds E.O.R. (1996). Lactate, *N*-acetylaspartate, choline and creatine concentrations, and spin-spin relaxation in thalamic and occipito-parietal regions of developing human brain. Magn. Reson. Med..

[9] Pouwels P.J.W., Brockmann K., Kruse B., Wilken B., Wick M., Hanefeld F., Frahm J. (1999). Regional Age Dependence of Human Brain Metabolites from Infancy to Adulthood as Detected by Quantitative Localized Proton MRS. Pediatr. Res..

[10] Kreis R., Hofmann L., Kuhlmann B., Boesch C., Bossi E., Hüppi P. (2002). Brain metabolite composition during early human brain development as measured by quantitative in vivo ^1^H magnetic resonance spectroscopy. Magn. Reson. Med..

[11] Blüml S., Wisnowski J.L., Nelson M.D., Paquette L., Gilles F.H., Kinney H.C., Panigrahy A. (2012). Metabolic Maturation of the Human Brain from Birth through Adolescence: Insights from In Vivo Magnetic Resonance Spectroscopy. Cereb. Cortex.

[12] Degnan A.J., Ceschin R., Lee V., Schmithorst V.J., Blüml S., Panigrahy A. (2014). Early Metabolic Development of Posteromedial cortex and Thalamus in Humans Using in vivo Quantitative MR Spectroscopy. J. Comp. Neurol..

[13] Bjartmar C., Battistuta J., Terada N., DuPree E., Trapp B.D. (2001). *N*-acetylaspartate is an axon-specific marker of mature white matter in vivo: A biochemical and immunohistochemical study on the rat optic nerve. Ann. Neurol..

[14] Burri R., Steffen C., Herschkowitz N. (1991). *N*-acetyl-l-aspartate is a major source of acetyl groups for lipid synthesis during rat brain development. Dev. Neurosci..

[15] Baslow M.H. (2000). Functions of *N*-acetyl-l-aspartate and *N*-acetyl-l-aspartylglutamate in the vertebrate brain: Role in glial cell-specific signaling. J. Neurochem..

[16] Moffett J.R., Ross B., Arun P., Madhavarao C.N., Namboodiri A.M. (2007). *N*-Acetylaspartate in the CNS: From neurodiagnostics to neurobiology. Prog. Neurobiol..

[17] Walker J.B. (1979). Creatine: Biosynthesis, regulation, and function. Adv. Enzymol. Relat. Areas Mol. Biol..

[18] Erecinska M., Silver I.A. (1990). Metabolism and role of glutamate in mammalian brain. Prog. Neurobiol..

[19] Thurston J.H., Sherman W.R., Hauhart R.E., Kloepper R.F. (1989). myo-inositol: A newly identified nonnitrogenous osmoregulatory molecule in mammalian brain. Pediatr. Res..

[20] Lien Y.H., Shapiro J.I., Chan L. (1990). Effects of hypernatremia on organic brain osmoles. J. Clin. Investig..

[21] Brand A., Richter-Landsberg C., Leibfritz D. (1993). Multinuclear NMR studies on the energy metabolism of glial and neuronal cells. Dev. Neurosci..

[22] Isaacks R.E., Bender A.S., Kim C.Y., Prieto N.M., Norenberg M.D. (1994). Osmotic regulation of myo-inositol uptake in primary astrocyte cultures. Neurochem. Res..

[23] Berry G.T. (2011). Is prenatal myo-inositol deficiency a mechanism of CNS injury in galactosemia?. J. Inherit. Metab. Dis..

[24] Perry T.L., Hansen S., Berry K., Mok C., Lesk D. (1971). Free amino acids and related compounds in biopsies of human brain. J. Neurochem..

[25] Kinney H.C., Karthigasan J., Borenshteyn N.I., Flax J.D., Kirschner D.A. (1994). Myelination in the developing human brain: Biochemical correlates. Neurochem. Res..

[26] Ackerstaff E., Glunde K., Bhujwalla Z.M. (2003). Choline phospholipid metabolism: A target in cancer cells?. J. Cell. Biochem..

[27] Blüml S., Wisnowski J.L., Nelson M.D., Paquette L., Panigrahy A. (2014). Metabolic maturation of white matter is altered in preterm infants. PLoS ONE.

[28] Ostrom Q.T., Gittleman H., Fulop J., Liu M., Blanda R., Kromer C., Wolinsky Y., Kruchko C., Barnholtz-Sloan J.S. (2015). CBTRUS Statistical Report: Primary Brain and Central Nervous System Tumors Diagnosed in the United States in 2008–2012. Neuro-Oncology.

[29] Gurney J., Smith M., Bunin G., Ries L., Smith M., Gurney J., Linet M., Tamra T., Young J. (1999). CNS and miscellaneous intracranial and intraspinal neoplasms. Cancer Incidence and Survival among Children and Adolescents: United States SEER Program 1975–1995.

[30] Wang Z., Sutton L.N., Cnaan A., Haselgrove J.C., Rorke L.B., Zhao H., Bilaniuk L.T., A Zimmerman R. (1995). Proton MR spectroscopy of pediatric cerebellar tumors. AJNR Am. J. Neuroradiol..

[31] Sutton L.N., Wang Z.J., Wehrli S.L., Marwaha S., Molloy P., Phillips P.C., Zimmerman R.A. (1997). Proton Spectroscopy of Suprasellar Tumors in Pediatric Patients. Neurosurgery.

[32] Arle J.E., Morriss C., Wang Z., Zimmerman R.A., Phillips P.G., Sutton L.N. (1997). Prediction of posterior fossa tumor type in children by means of magnetic resonance image properties, spectroscopy, and neural networks. J. Neurosurg..

[33] Tzika A.A., Astrakas L.G., Zarifi M.K., Zurakowski D., Poussaint T.Y., Goumnerova L., Tarbell N.J., Black P.M. (2004). Spectroscopic and perfusion magnetic resonance imaging predictors of progression in pediatric brain tumors. Cancer.

[34] A Lazareff J., Olmstead C., Bockhorst K.H., Alger J.R. (1996). Proton magnetic resonance spectroscopic imaging of pediatric low-grade astrocytomas. Child’s Nerv. Syst..

[35] Peet A.C., Lateef S., MacPherson L., Natarajan K., Sgouros S., Grundy R.G. (2006). Short echo time 1 H magnetic resonance spectroscopy of childhood brain tumours. Child’s Nerv. Syst..

[36] Davies N.P., Wilson M., Harris L.M., Natarajan K., Lateef S., Macpherson L., Sgouros S., Grundy R.G., Arvanitis T.N., Peet A.C. (2008). Identification and characterisation of childhood cerebellar tumours by in vivo proton MRS. NMR Biomed..

[37] Panigrahy A., Krieger M., Gonzalez-Gomez I., Liu X., McComb J., Finlay J., Nelson M., Gilles F., Blüml S. (2006). Quantitative Short Echo Time ^1^H-MR Spectroscopy of Untreated Pediatric Brain Tumors: Preoperative Diagnosis and Characterization. Am. J. Neuroradiol..

[38] Shiroishi M.S., Panigrahy A., Moore K.R., Nelson M.D., Gilles F.H., González-Gómez I., Blüml S. (2015). Combined MRI and MRS improves pre-therapeutic diagnoses of pediatric brain tumors over MRI alone. Neuroradiology.

[39] Tamrazi B., Nelson M.D., Blüml S. (2016). MRS of pilocytic astrocytoma: The peak at 2 ppm may not be NAA. Magn. Reson. Med..

[40] Jones C., Karajannis M.A., Jones D.T.W., Kieran M.W., Monje M., Baker S.J., Becher O.J., Cho Y.-J., Gupta N., Hawkins C. (2016). Pediatric high-grade glioma: Biologically and clinically in need of new thinking. Neuro-Oncology.

[41] Blüml S., Margol A.S., Sposto R., Kennedy R.J., Robison N.J., Vali M., Hung L.T., Muthugounder S., Finlay J.L., Erdreich-Epstein A. (2015). Molecular subgroups of medulloblastoma identification using noninvasive magnetic resonance spectroscopy. Neuro Oncol..

[42] Harris L.M., Davies N., MacPherson L., Lateef S., Natarajan K., Brundler M.-A., Sgouros S., English M.W., Arvanitis T., Grundy R.G. (2008). Magnetic resonance spectroscopy in the assessment of pilocytic astrocytomas. Eur. J. Cancer.

[43] Opstad K., Provencher S., Bell B., Griffiths J., Howe F. (2003). Detection of elevated glutathione in meningiomas by quantitative in vivo ^1^H MRS. Magn. Reson. Med..

[44] Gill S.S., Thomas D.G., Van Bruggen N., Gadian D.G., Peden C.J., Bell J.D., Cox I.J., Menon D.K., Iles R.A., Bryant D.J. (1990). Proton MR spectroscopy of intracranial tumours: In vivo and in vitro studies. J. Comput. Assist. Tomogr..

[45] Shimizu H., Kumabe T., Shirane R., Yoshimoto T. (2000). Correlation between Choline Level Measured by Proton MR Spectroscopy and Ki-67 Labeling Index in Gliomas. Am. J. Neuroradiol..

[46] Clymer J., Kieran M.W. (2018). The Integration of Biology Into the Treatment of Diffuse Intrinsic Pontine Glioma: A Review of the North American Clinical Trial Perspective. Front. Oncol..

[47] Pan E., Prados M., Gupta N., Haas-Kogen D., Banerjee A. Pediatric CNS Tumors.

[48] Yoshimura J., Onda K., Tanaka R., Takahashi H. (2003). Clinicopathological Study of Diffuse Type Brainstem Gliomas: Analysis of 40 Autopsy Cases. Neurol. Med.-Chir..

[49] Panigrahy A., Nelson M.D., Finlay J.L., Sposto R., Krieger M.D., Gilles F.H., Blüml S. (2008). Metabolism of diffuse intrinsic brainstem gliomas in children. Neuro-Oncology.

[50] Seymour Z.A., Panigrahy A., Finlay J.L., Nelson M.D., Bluml S. (2008). Citrate in pediatric CNS tumors?. AJNR Am. J. Neuroradiol..

[51] Blüml S., Panigrahy A., Laskov M., Dhall G., Krieger M.D., Nelson M.D., Finlay J.L., Gilles F.H. (2011). Elevated citrate in pediatric astrocytomas with malignant progression. Neuro-Oncology.

[52] Davies N.P., Wilson M., Natarajan K., Sun Y., MacPherson L., Brundler M.A., Arvanitis T.N., Grundy R.G., Peet A.C. (2010). Non-invasive detection of glycine as a biomarker of malignancy in childhood brain tumours using in-vivo ^1^H MRS at 1.5 tesla confirmed by ex-vivo high-resolution magic-angle spinning NMR. NMR Biomed..

[53] Carapella C.M., Carpinelli G., Knijn A., Raus L., Caroli F., Podo F. (1997). Potential Role of in vitro ^1^H Magnetic Resonance Spectroscopy in the Definition of Malignancy Grading of Human Neuroepithelial Brain Tumours. Acta Neurochir. Suppl..

[54] Tzika A.A., Righi V., Andronesi O.C., Mintzopoulos D., Black P.M. (2009). High-resolution magic angle spinning magnetic resonance spectroscopy detects glycine as a biomarker in brain tumors. Int. J. Oncol..

[55] Louis D.N., Perry A., Wesseling P., Brat D.J., Cree I.A., Figarella-Branger D., Hawkins C., Ng H.K., Pfister S.M., Reifenberger G. (2021). The 2021 WHO Classification of Tumors of the Central Nervous System: A summary. Neuro-Oncology.

[56] A Northcott P., Dubuc A.M., Pfister S., Taylor M.D. (2012). Molecular subgroups of medulloblastoma. Expert Rev. Neurother..

[57] Tamrazi B., Venneti S., Margol A., Hawes D., Cen S., Nelson M., Judkins A., Biegel J., Blüml S. (2019). Pediatric Atypical Teratoid/Rhabdoid Tumors of the Brain: Identification of Metabolic Subgroups Using In Vivo ^1^H-MR Spectroscopy. Am. J. Neuroradiol..

[58] Panwalkar P., Tamrazi B., Dang D., Chung C., Sweha S., Natarajan S.K., Pun M., Bayliss J., Ogrodzinski M.P., Pratt D. (2021). Targeting integrated epigenetic and metabolic pathways in lethal childhood PFA ependymomas. Sci. Transl. Med..

[59] Lawn J., Shibuya K., Stein C. (2005). No cry at birth: Global estimates of intrapartum stillbirths and intrapartum-related neonatal deaths. Bull. World Health Organ..

[60] Barkovich A.J., Hajnal B.L., Vigneron D., Sola A., Partridge J.C., Allen F., Ferriero D.M. (1998). Prediction of neuromotor outcome in perinatal asphyxia: Evaluation of MR scoring systems. AJNR Am. J. Neuroradiol..

[61] Miller S., Ramaswamy V., Michelson D., Barkovich A.J., Holshouser B., Wycliffe N., Glidden D., Deming D., Partridge J.C., Wu Y.W. (2005). Patterns of brain injury in term neonatal encephalopathy. J. Pediatr..

[62] Groenendaal F., Veenhoven R.H., van der Grond J., Jansen G.H., Witkamp T.D., de Vries L.S. (1994). Cerebral Lactate and *N*-Acetyl-Aspartate/Choline Ratios in Asphyxiated Full-Term Neonates Demonstrated In Vivo Using Proton Magnetic Resonance Spectroscopy. Pediatr. Res..

[63] Alderliesten T., De Vries L.S., Benders M.J.N.L., Koopman C., Groenendaal F. (2011). MR Imaging and Outcome of Term Neonates with Perinatal Asphyxia: Value of Diffusion-weighted MR Imaging and H MR Spectroscopy. Radiology.

[64] Parmentier C.E.J., de Vries L.S., Groenendaal F. (2022). Magnetic Resonance Imaging in (Near-)Term Infants with Hypoxic-Ischemic Encephalopathy. Diagnostics.

[65] Miller S.P., Newton N., Ferriero D.M., Partridge J.C., Glidden D.V., Barnwell A., A Chuang N., Vigneron D.B., Barkovich A.J. (2002). Predictors of 30-Month Outcome after Perinatal Depression: Role of Proton MRS and Socioeconomic Factors. Pediatr. Res..

[66] Azzopardi D., Edwards A.D. (2010). Magnetic resonance biomarkers of neuroprotective effects in infants with hypoxic ischemic encephalopathy. Semin. Fetal Neonatal Med..

[67] Cheong J., Cady E., Penrice J., Wyatt J., Cox I., Robertson N. (2006). Proton MR Spectroscopy in Neonates with Perinatal Cerebral Hypoxic-Ischemic Injury: Metabolite Peak-Area Ratios, Relaxation Times, and Absolute Concentrations. Am. J. Neuroradiol..

[68] Barkovich A.J., Baranski K., Vigneron D., Partridge J.C., Hallam D.K., Hajnal B.L., Ferriero D.M. (1999). Proton MR Spectroscopy for the Evaluation of Brain Injury in Asphyxiated, Term Neonates. Am. J. Neuroradiol..

[69] Mitra S., Kendall G.S., Bainbridge A., Sokolska M., Dinan M., Uria-Avellanal C., Price D., McKinnon K., Gunny R., Huertas-Ceballos A. (2018). Proton magnetic resonance spectroscopy lactate/*N*-acetylaspartate within 2 weeks of birth accurately predicts 2-year motor, cognitive and language outcomes in neonatal encephalopathy after therapeutic hypothermia. Arch. Dis. Child.-Fetal Neonatal Ed..

[70] Aida N. (2022). ^1^H-MR Spectroscopy of the Early Developmental Brain, Neonatal Encephalopathies, and Neurometabolic Disorders. Magn. Reson. Med. Sci..

[71] Thayyil S., Chandrasekaran M., Taylor A., Bainbridge A., Cady E.B., Chong W.K.K., Murad S., Omar R.Z., Robertson N.J. (2010). Cerebral Magnetic Resonance Biomarkers in Neonatal Encephalopathy: A Meta-analysis. Pediatrics.

[72] Shanmugalingam S., Thornton J.S., Iwata O., Bainbridge A., O’Brien F.E., Priest A.N., Ordidge R.J., Cady E.B., Wyatt J.S., Robertson N.J. (2006). Comparative Prognostic Utilities of Early Quantitative Magnetic Resonance Imaging Spin-Spin Relaxometry and Proton Magnetic Resonance Spectroscopy in Neonatal Encephalopathy. Pediatrics.

[73] Kreis R., Arcinue E., Ernst T., Shonk T.K., Flores R., Ross B.D. (1996). Hypoxic encephalopathy after near-drowning studied by quantitative ^1^H-magnetic resonance spectroscopy. J. Clin. Investig..

[74] Cady E.B., Lorek A., Penrice J., Reynolds E.O., Iles R.A., Burns S.P., Coutts G.A., Cowan F.M. (1994). Detection of propan-1,2-diol in neonatal brain by in vivo proton magnetic resonance spectroscopy. Magn. Reson. Med..

[75] Whitehead M.T., Lai L.M., Blüml S. (2022). Clinical ^1^H MRS in childhood neurometabolic diseases—Part 1: Technique and age-related normal spectra. Neuroradiology.

[76] Cecil K.M., Lindquist D.M., Bluml S., Panigrahy A. (2013). Leukodystrophies. MR Spectroscopy of Pediatric Brain Disorders.

[77] Cecil K.M., Lindquist D.M., Bluml S., Panigrahy A. (2013). Metabolic Disorders. MR Spectroscopy of Pediatric Brain Disorders.

[78] Wilken B., Dechent P., Hanefeld F., Frahm J. (2008). Proton MRS of a child with Sandhoff disease reveals elevated brain hexosamine. Eur. J. Paediatr. Neurol..

[79] Paul A.R., Adamo M.A. (2014). Non-accidental trauma in pediatric patients: A review of epidemiology, pathophysiology, diagnosis and treatment. Transl. Pediatr..

[80] Keenan H.T., Runyan D.K., Marshall S.W., Nocera M.A., Merten D.F., Sinal S.H. (2003). A population-based study of inflicted traumatic brain injury in young children. JAMA.

[81] Theodore A.D., Chang J.J., Runyan D.K., Hunter W.M., Bangdiwala S.I., Agans R. (2005). Epidemiologic Features of the Physical and Sexual Maltreatment of Children in the Carolinas. Pediatrics.

[82] Kay T., Harrington D.E.R.A., Anderson T., Berrol S., Cicerone K. (1993). Definition of mild traumatic brain injury. J. Head Trauma Rehabil..

[83] Arbogast K.B., Curry A., Pfeiffer M.R., Zonfrillo M., Haarbauer-Krupa J., Breiding M.J., Coronado V.G., Master C. (2016). Point of Health Care Entry for Youth With Concussion Within a Large Pediatric Care Network. JAMA Pediatr..

[84] Meehan W.P., Bachur R.G. (2009). Sport-related concussion. Pediatrics.

[85] Field M., Collins M.W., Lovell M.R., Maroon J. (2003). Does age play a role in recovery from sports-related concussion? A comparison of high school and collegiate athletes. J. Pediatr..

[86] Makoroff K.L., Cecil K.M., Caré M., Ball W.S. (2005). Elevated lactate as an early marker of brain injury in inflicted traumatic brain injury. Pediatr. Radiol..

[87] Ashwal S., A Holshouser B., Shu S.K., Simmons P.L., Perkin R.M., Tomasi L.G., Knierim D.S., Sheridan C., Craig K., Andrews G.H. (2000). Predictive value of proton magnetic resonance spectroscopy in pediatric closed head injury. Pediatr. Neurol..

[88] Aaen G.S., Holshouser B.A., Sheridan C., Colbert C., McKenney M., Kido D., Ashwal S. (2010). Magnetic Resonance Spectroscopy Predicts Outcomes for Children With Nonaccidental Trauma. Pediatrics.

[89] Haseler L.J., Arcinue E., Danielsen E.R., Bluml S., Ross B.D. (1997). Evidence From Proton Magnetic Resonance Spectroscopy for a Metabolic Cascade of Neuronal Damage in Shaken Baby Syndrome. Pediatrics.

[90] A Holshouser B., Ashwal S., Luh G.Y., Shu S., Kahlon S., Auld K.L., Tomasi L.G., Perkin R.M., Hinshaw D.B. (1997). Proton MR spectroscopy after acute central nervous system injury: Outcome prediction in neonates, infants, and children. Radiology.

[91] Ross B.D., Ernst T., Kreis R., Haseler L.J., Bayer S., Danielsen E., Bluml S., Shonk T., Mandigo J.C., Caton W. (1998). ^1^H MRS in acute traumatic brain injury. J. Magn. Reson. Imaging.

[92] Holshouser B.A., Ashwal S., Shu S., Hinshaw D.B. (2000). Proton MR spectroscopy in children with acute brain injury: Comparison of short and long echo time acquisitions. J. Magn. Reson. Imaging.

[93] Friedman S., Brooks W., Jung R., Chiulli S., Sloan J., Montoya B., Hart B., Yeo R. (1999). Quantitative proton MRS predicts outcome after traumatic brain injury. Neurology.

[94] Holshouser B.A., Tong K.A., Ashwal S. (2005). Proton MR Spectroscopic Imaging Depicts Diffuse Axonal Injury in Children with Traumatic Brain Injury. Am. J. Neuroradiol..

[95] Govindaraju V., Gauger G.E., Manley G.T., Ebel A., Meeker M., Maudsley A.A. (2004). Volumetric Proton Spectroscopic Imaging of Mild Traumatic Brain Injury. Am. J. Neuroradiol..

[96] Brooks W.M., Stidley C.A., Petropoulos H., Jung R.E., Weers D.C., Friedman S., Barlow M.A., Sibbitt W., Yeo R.A. (2000). Metabolic and Cognitive Response to Human Traumatic Brain Injury: A Quantitative Proton Magnetic Resonance Study. J. Neurotrauma.

[97] Gasparovic C., Arfai N., Smid N., Feeney D.M. (2001). Decrease and Recovery of *N*-Acetylaspartate/Creatine in Rat Brain Remote from Focal Injury. J. Neurotrauma.

[98] Schuhmann M.U., Stiller D., Skardelly M., Thomas S., Samii M., Brinker T. (2002). Long-Time in-Vivo Metabolic Monitoring Following Experimental Brain Contusion Using Proton Magnetic Resonance Spectroscopy. Acta Neurochir. Suppl..

[99] Cecil K.M., Hills E.C., Sandel M.E., Smith D.H., McIntosh T.K., Mannon L.J., Sinson G.P., Bagley L.J., Grossman R.I., Lenkinski R.E. (1998). Proton magnetic resonance spectroscopy for detection of axonal injury in the splenium of the corpus callosum of brain-injured patients. J. Neurosurg..

[100] Pal D., Bhattacharyya A., Husain M., Prasad K., Pandey C., Gupta R. (2009). In Vivo Proton MR Spectroscopy Evaluation of Pyogenic Brain Abscesses: A Report of 194 Cases. Am. J. Neuroradiol..

[101] Lai P.-H., Hsu S.-S., Ding S.-W., Ko C.-W., Fu J.-H., Weng M.-J., Yeh L.-R., Wu M.-T., Liang H.-L., Chen C.-K. (2007). Proton magnetic resonance spectroscopy and diffusion-weighted imaging in intracranial cystic mass lesions. Surg. Neurol..

[102] Luthra G., Parihar A., Nath K., Jaiswal S., Prasad K., Husain N., Husain M., Singh S., Behari S., Gupta R. (2007). Comparative Evaluation of Fungal, Tubercular, and Pyogenic Brain Abscesses with Conventional and Diffusion MR Imaging and Proton MR Spectroscopy. Am. J. Neuroradiol..

[103] Gupta R.K., Jain K.K., Mittal S.K., Kumar S. (2007). Imaging features of central nervous system fungal infections. Neurol. India.

[104] Ferraz-Filho J.R., Santana-Netto P.V., Rocha-Filho J.A., Sgnolf A., Mauad F., Sanches R.A. (2009). Application of magnetic resonance spectroscopy in the differentiation of high-grade brain neoplasm and inflammatory brain lesions. Arq. Neuro-Psiquiatr..

[105] Keller M.A., Venkatraman T.N., Thomas A., Deveikis A., LoPresti C., Hayes J., Berman N., Walot I., Padilla S., Johnston-Jones J. (2004). Altered neurometabolite development in HIV-infected children: Correlation with neuropsychological tests. Neurology.

[106] van der Voorn J.P., Pouwels P.J., Vermeulen R.J., Barkhof F., van der Knaap M.S. (2009). Quantitative MR imaging and spectroscopy in congenital cytomegalovirus infection and periventricular leukomalacia suggests a comparable neuropathological substrate of the cerebral white matter lesions. Neuropediatrics.

[107] Takanashi J.-I., Sugita K., Ishii M., Aoyagi M., Niimi H. (1997). Longitudinal MR imaging and proton MR spectroscopy in herpes simplex encephalitis. J. Neurol. Sci..

[108] Cecil K.M., Jones B.V., Williams S., Hedlund G.L. (2000). CT, MRI and MRS of Epstein-Barr virus infection: Case report. Neuroradiology.

[109] Cecil K.M., Lindquist D.M. (2013). Infection and Encephalitis. MR Spectroscopy of Pediatric Brain Disorders.

[110] Mader I., Wolff M., Nägele T., Niemann G., Grodd W., Küker W. (2005). MRI and proton MR spectroscopy in acute disseminated encephalomyelitis. Child’s Nerv. Syst..

[111] Seo H.-E., Hwang S.-K., Choe B.H., Cho M.-H., Park S.-P., Kwon S. (2010). Clinical Spectrum and Prognostic Factors of Acute Necrotizing Encephalopathy in Children. J. Korean Med. Sci..

[112] Gupta R.K., Roy R., Dev R., Husain M., Poptani H., Pandey R., Kishore J., Bhaduri A.P. (1996). Finger printing of Mycobacterium tuberculosis in patients with intracranial tuberculomas by using in vivo, ex vivo, and in vitro magnetic resonance spectroscopy. Magn. Reson. Med..

[113] Gupta R.K., Husain M., Vatsal D.K., Kumar R., Chawla S., Husain N. (2002). Comparative evaluation of magnetization transfer MR imaging and in-vivo proton MR spectroscopy in brain tuberculomas. Magn. Reson. Imaging.

[114] Malhotra H., Jain K., Agarwal A., Singh M., Yadav S., Husain M., Krishnani N., Gupta R. (2008). Characterization of tumefactive demyelinating lesions using MR imaging and in-vivo proton MR spectroscopy. Mult. Scler. J..

[115] Cianfoni A., Niku S., Imbesi S. (2007). Metabolite Findings in Tumefactive Demyelinating Lesions Utilizing Short Echo Time Proton Magnetic Resonance Spectroscopy. Am. J. Neuroradiol..

[116] Saindane A.M., Cha S., Law M., Xue X., Knopp E.A., Zagzag D. (2002). Proton MR Spectroscopy of Tumefactive Demyelinating Lesions. Am. J. Neuroradiol..

[117] Urenjak J., Williams S.R., Gadian D.G., Noble M. (1992). Specific expression of *N*-acetylaspartate in neurons, oligodendrocyte-type-2 astrocyte progenitors, and immature oligodendrocytes in vitro. J. Neurochem..

[118] Signoretti S., Marmarou A., Tavazzi B., Lazzarino G., Beaumont A., Vagnozzi R. (2001). *N*-Acetylaspartate Reduction as a Measure of Injury Severity and Mitochondrial Dysfunction Following Diffuse Traumatic Brain Injury. J. Neurotrauma.

[119] Varho T., Komu M., Sonninen P., Lähdetie J., Holopainen I.E. (2005). Quantitative ^1^H MRS and MRI Volumetry Indicate Neuronal Damage in the Hippocampus of Children with Focal Epilepsy and Infrequent Seizures. Epilepsia.

[120] Miller E., Widjaja E., Bluml S., Panigrahy A. (2013). Magnetic Resonance Spectroscopy in Epilepsy. MR Spectroscopy of Pediatric Brain Disorders.

[121] Najm I.M., Wang Y., Hong S.C., Luders H.O., Ng T.C., Comair Y.G. (1997). Temporal Changes in Proton MRS Metabolites After Kainic Acid-Induced Seizures in Rat Brain. Epilepsia.

[122] Baslow M.H. (2002). Evidence supporting a role for *N*-acetyl-l-aspartate as a molecular water pump in myelinated neurons in the central nervous system: An analytical review. Neurochem. Int..

[123] Najm I.M., Wang Y., Shedid D., Luders H.O., Ng T.C., Comair Y.G. (1998). MRS metabolic markers of seizures and seizure-induced neuronal damage. Epilepsia.

[124] Woermann F.G., McLean M.A., Bartlett P.A., Parker G.J., Barker G.J., Duncan J.S. (1999). Short echo time single-voxel ^1^H magnetic resonance spectroscopy in magnetic resonance imaging-negative temporal lobe epilepsy: Different biochemical profile compared with hippocampal sclerosis. Ann. Neurol..

[125] Simister R.J., McLean M.A., Barker G.J., Duncan J.S. (2003). A Proton Magnetic Resonance Spectroscopy Study of Metabolites in the Occipital Lobes in Epilepsy. Epilepsia.

[126] Sherwin A., Robitaille Y., Quesney F., Olivier A., Villemure J., Leblanc R., Feindel W., Andermann E., Gotman J., Ethier R. (1988). Excitatory amino acids are elevated in human epileptic cerebral cortex. Neurology.

[127] Petroff O.A., Pleban L.A., Spencer D.D. (1995). Symbiosis between in vivo and in vitro NMR spectroscopy: The creatine, *N*-acetylaspartate, glutamate, and GABA content of the epileptic human brain. Magn. Reson. Imaging.

[128] Pfund Z., Chugani D.C., Juhász C., Muzik O., Chugani H.T., Wilds I.B., Seraji-Bozorgzad N., Moore G.J. (2000). Evidence for Coupling between Glucose Metabolism and Glutamate Cycling Using FDG PET and ^1^H Magnetic Resonance Spectroscopy in Patients with Epilepsy. J. Cereb. Blood Flow Metab..

[129] Seymour K.J., Bluml S., Sutherling J., Sutherling W., Ross B.D. (1999). Identification of cerebral acetone by ^1^H-MRS in patients with epilepsy controlled by ketogenic diet. Magma.

[130] Horska A., Mahone E.M., Bluml S., Panigrahy A. (2013). ^1^H Magnetic Resonance Spectroscopy of the Brain During Adolescence: Normal Brain Development and Neuropsychiatric Disorders. MR Spectroscopy of Pediatric Brain Disorders.

[131] Levitt J.G., O’Neill J., Alger J.R., Bluml S., Panigrahy A. (2013). Magnetic Resonance Spectroscopy Studies of Autistic Spectrum Disorders. MR Spectroscopy of Pediatric Brain Disorders.

[132] O’Neill J., Levitt J.G., Alger J.R., Bluml S., Panigrahy A. (2013). Magnetic Resonance Spectroscopy Studies of Attention Deficit Hyperactivity Disorder. MR Spectroscopy of Pediatric Brain Diseases.

